# Associating broad and clinically defined polygenic scores for depression with depression-related phenotypes

**DOI:** 10.1038/s41598-023-33645-7

**Published:** 2023-04-21

**Authors:** John E. McGeary, Chelsie E. Benca-Bachman, Victoria A. Risner, Christopher G. Beevers, Brandon E. Gibb, Rohan H. C. Palmer

**Affiliations:** 1 Providence Veterans Affairs Medical Center, Providence, RI USA; 2 Behavioral Genetics of Addiction Laboratory, Department of Psychology, Emory University, 36 Eagle Row, Atlanta, GA 30322 USA; 3 Department of Psychology, University of Texas at Austin, Austin, TX USA; 4 Department of Psychology State, University of New York at Binghamton, Binghamton, NY USA

**Keywords:** Genetics, Psychology

## Abstract

Twin studies indicate that 30–40% of the disease liability for depression can be attributed to genetic differences. Here, we assess the explanatory ability of polygenic scores (PGS) based on broad- (PGS_BD_) and clinical- (PGS_MDD_) depression summary statistics from the UK Biobank in an independent sample of adults (N = 210; 100% European Ancestry) who were extensively phenotyped for depression and related neurocognitive traits (e.g., rumination, emotion regulation, anhedonia, and resting frontal alpha asymmetry). The UK Biobank-derived PGS_BD_ had small associations with MDD, depression severity, anhedonia, cognitive reappraisal, brooding, and suicidal ideation but only the association with suicidal ideation remained statistically significant after correcting for multiple comparisons. Similarly small associations were observed for the PGS_MDD_ but none remained significant after correcting for multiple comparisons. These findings provide important initial guidance about the expected effect sizes between current UKB PGSs for depression and depression-related neurocognitive phenotypes.

## Introduction

There are clear genetic influences on depression risk^1^ and heritability estimates from twin studies suggest that 30–40% of risk for depression can be attributed to genetic influences^2^. Although initial genome-wide association studies (GWAS) provided inconsistent evidence^3–5^, a recent, well-powered GWAS of depression has identified a more reliable set of genetic associations. Howard and colleagues found 102 independent variants in a discovery sample (*N* = 807,553) of which 87 were replicated in the validation sample (*N* = 1,306,354)^6^. Reliable findings in depression GWAS studies usher in an era of possibility wherein the identification of the specific heritable genetic variants may lead to novel insights for treatment or prevention.

In addition to identifying reliable single nucleotide polymorphisms (SNPs) associated with a particular phenotype, large, GWASs allow for the calculation of polygenic scores (PGS) that aggregate individual small genetic effects to summarize a person’s lifetime genetic risk of disease. These scores are critical for understanding the clinical importance of genetic influences to psychiatric disorders, such as depression, since the individual effects of commonly occurring polymorphisms are typically too subtle to be meaningful in isolation.

To maximize the value of genetic findings for depression research, the phenotypes used to glean reliable findings must be carefully considered. Phenotypes used in GWAS are typically limited in scope (by design) to simplified definitions that indicate the presence or absence of disease in order to obtain large samples. For instance, the broad depression phenotype in the UK Biobank (UKB), a study assessing a variety of health characteristics in a prospective population-based cohort of over 500,000 men and women from the UK, was based on endorsement (yes/no) of a single item, “Have you ever seen a general practitioner for nerves, anxiety, tension or depression?”^6,7^. Prior work has shown a strong genetic correlation (*r*G = 0.86, SE = 0.05) between self-reported definitions of depression and clinically diagnosed major depressive disorder (MDD), with the former being easier to obtain^8^.

On one hand it is important to define the nomological network^9^ of constructs or detailed phenotypes (e.g., negative cognition; specific depression symptoms) associated with genetic variants initially identified by GWAS to better understand how genetic variation influences specific traits or features and in turn how these features impact the manifestation of the broader disorder. On the other hand, considering that sample sizes in the 100,000 s are required for a GWAS study it can be challenging to engage in detailed phenotyping of depression-relevant constructs, such as electroencephalography or suicidal ideation. However, the potential of this additional effort has been highlighted by the important etiological insights in SCZ obtained by the phenotypic annotation approach^10^. For instance, SNPs for schizophrenia identified by GWAS have been associated with known risk factors and correlates of the disorder, including neighborhood disadvantage^11^, illicit drug use^12^, and creativity^13^. Among people initially hospitalized for psychosis, PGSs for schizophrenia predict the occurrence of more severe negative symptoms, lower global assessment of functioning, more impaired cognition, and the eventual development of a schizophrenia spectrum disorder across a 20-year post-hospitalization follow-up period^14^.

It is currently unknown whether depression-relevant endophenotypes are associated with the SNPs identified by large scale GWAS. Prior work suggests that the effects may be small. For instance, polygenic scores derived from the Psychiatric Genomics Consortium genome-wide association study of MDD explained less than 1% of the variance in depression symptom severity in an independent sample^15^ and 1.1% of the variance MDD status in a case–control study^16^, which is an improvement from a prior GWAS^17^. A recent study by Mitchell and colleagues (2021) associated depression PGSs with clinical features that extend beyond diagnosis (e.g., age of first depressive episode, 2 or more depressive episodes)^18^ in a clinical sample. However, the association between GWAS-derived PGSs and depression-related neurocognitive phenotypes has not been thoroughly examined to date.

Should a large-scale GWAS-derived PGS for depression reliably index one of these intermediate phenotypes, but not others, it could help identify mechanisms that link SNPs with depression risk, indexing specific endophenotypes of depression and potentially inform the future development of personalized/targeted treatment efforts. Given the heterogeneity of depression^19^, if a GWAS-derived PGS does not predict a particular intermediate phenotype, it might suggest that the PGS is indexing one particular aspect of depression over another. Accordingly, depression relevant PGSs should be used to determine whether they provide additional insights into the genetic basis of established neurocognitive phenotypes.

The current study examined associations between polygenic scores derived from a recent GWAS of depression in the UKB^8^ and a broad array of neurocognitive phenotypes associated with depression collected in an independent sample of 210 adults who ranged in depression severity. In addition to diagnoses and symptoms of depression, we also examined the relative utility of broadly- versus clinically-defined PGS in predicting depression-relevant phenotypes including self-reported rumination, emotion regulation, anhedonia, and resting frontal alpha asymmetry. These phenotypes are highly relevant to depression^20–22^, can be measured with good reliability^23–26^, and appear to be heritable to varying degrees^27–32^. Thus, they are promising candidates for examining associations with polygenic scores for depression.

## Results

Table 1 provides descriptive information for the depression-relevant phenotypes. Participants were mostly female, in their mid-20 s. Much of the sample had experienced a past episode of depression (60.5%). Nearly a third (27.8%) of participants met criteria for current MDD and scores on the BDI-II ranged from 0 to 57 (*M* = 17.98, *SD* = 11.55). A third of participants endorsed having current suicidal ideation or wishes. All other outcomes had sufficient variability to warrant exploration PGSs could be associated with variability in the phenotype. To increase normality in the distributions, multivariate regressions with age and sex as predictors were run to create standardized residuals for each depression-related phenotype. Then the subsequent multivariable regression analyses reported in Table 2 were run.

**Table 1:** Descriptive statistics of depression relevant outcomes and covariates.

Phenotype	N	Mean	SD	Min	Max
Age	218	23.29	4.88	18.00	36.00
Sex (% female)	218	66.06	N/A	N/A	N/A
MDD—current (% yes)	216	27.78	N/A	N/A	N/A
BDI-II	218	17.98	11.55	00.00	57.00
Alpha asymmetry (f7/f8)	206	-0.06	0.19	-0.76	0.61
ERQ cognitive reappraisal	218	25.73	7.62	6.00	42.00
ERQ suppression	218	15.36	5.49	4.00	27.00
SHAPS anhedonia	218	2.75	2.72	0.00	11.00
RRS brooding	218	11.49	3.67	5.00	20.00
BDI-II suicide item	217	0.34	0.52	0.00	3.00

*SD* standard deviation, *Min* minimum, *Max* maximum, *MDD* major depressive disorder, *BDI-II* beck depression inventory-II, *ERQ* emotion regulation questionnaire, *SHAPS* Snaith–Hamilton pleasure scale, *RRS* ruminative response scale, *N/A* not applicable.

**Table 2:** Polygenic scores (PGS) effect on depression relevant outcomes.

Phenotype	Broad depression PGS					
	β [95% CI]	*p* (unadj)	*p* (adj)	R^2^ Estimate	R^2^ SE	R^2^* p*
MDD (current)	0.18 [0.04, 0.31]	0.01	0.06	0.05	0.03	0.09
Beck depression inventory-II	0.17 [0.03, 0.31]	0.01	0.05	0.03	0.02	0.17
Alpha asymmetry (f7/f8)	0.11 [− 0.03, 0.24]	0.14	0.26	0.06	0.03	0.06
ERQ cognitive reappraisal	− 0.17 [− 0.31, − 0.04]	0.01	0.06	0.05	0.03	0.10
ERQ suppression	0.04 [− 0.09, 0.17]	0.56	1.00	0.07	0.04	0.03
SHAPS anhedonia	0.14 [0.01, 0.28]	0.04	0.11	0.04	0.03	0.15
RRS brooding	0.17 [0.04, 0.31]	0.01	0.06	0.04	0.03	0.12
BDI-II suicide item	0.20 [0.06, 0.33]	0	0.03	0.06	0.03	0.07

Phenotype	MDD Diagnosis PGS					
	β [95% CI]	*p* (unadj)	*p* (adj)	R^2^ Estimate	R^2^ SE	R^2^* p*
MDD (current)	0.15 [0.02, 0.29]	0.03	0.17	0.04	0.03	0.12
Beck depression inventory-II	0.15 [0.02, 0.29]	0.03	0.16	0.03	0.02	0.21
Alpha asymmetry (f7/f8)	− 0.09 [− 0.23, 0.05]	0.21	0.498	0.02	0.02	0.32
ERQ cognitive reappraisal	− 0.15 [− 0.28, − 0.02]	0.03	0.16	0.04	0.03	0.13
ERQ suppression	0.10 [− 0.03, 0.23]	0.15	0.46	0.08	0.04	0.03
SHAPS anhedonia	0.05 [− 0.08, 0.19]	0.44	0.68	0.02	0.02	0.29
RRS brooding	0.15 [0.01, 0.28]	0.03	0.14	0.04	0.03	0.16
BDI-II suicide item	0.04 [− 0.10, 0.17]	0.6	1.00	0.02	0.02	0.29

*ERQ* emotion regulation questionnaire, *SHAPS* Snaith–Hamilton pleasure scale, *RRS* ruminative response scale.

p(unadj) is the observed two-tailed, p-value uncorrected for multiple testing with correlated outcomes. p(adj) refers to the false discovery rate adjusted p-value for correlated outcomes.

### PGS_BD_ and PGS_MDD_ relations with symptoms and diagnoses of depression

Both broad (PGS_*BD*_) and clinical (PGS_*MDD*_) operationalization of polygenic risk for depression were associated with increased depression severity and MDD diagnosis (Table 2). Although none of the associations survived correction for multiple testing, PGS_*BD*_ corrected *p*-values for current MDD diagnosis and depression severity were marginally significant at 0.061 and 0.053, respectively.

### PGS_BD_ and PGS_MDD_ relations with depression-related phenotypes

Polygenic effects across depression-related phenotypes were mixed and varied by PGS (Table 2). Higher PGS_BD_ was associated with increased suicidal thoughts and ideation, brooding and anhedonia, and lower levels of cognitive reappraisal. A similar pattern was observed for the PGS_MDD_ (with the exception of anhedonia and suicidal ideation), although effect sizes were smaller than that of PGS_BD_ and did not survive correction for multiple testing. Neither PGS_BD_ nor PGS_MDD_ were associated with alpha asymmetry.

## Discussion

This study examined the utility of polygenic scores derived from GWAS of “broad-depression” (PGS_BD_) and MDD (PGS_MDD_) in an independent sample that had been characterized for eight depression-related phenotypes. These phenotypes included diagnostic and standardized measures of depression, electrophysiology, and cognitive assessments. Primary questions included: (a) whether the broad-depression PGS accounted for significant variance in depression-related phenotypes in a well-characterized adult sample, and (b) how a more focused MDD PGS performed in the same sample.

The PGS_BD_ yielded six suggestive findings (see Table 2), though only one phenotype, suicidal ideation, survived correction for multiple testing. This suggests that a broad depression PGS, though low in specificity for depression liability^33^, may have utility for some but not other depression-related phenotypes^18^. The pattern of findings may hint at the type of “depression” indexed by the items used to create the PGS in UKB. While more work needs to be done, the current pattern of results suggests that perhaps those who answer affirmatively to the question about seeking help for nerves, anxiety, tension, or depression might be more likely to: (1) have an MDD diagnosis; (2) have higher levels of depressive symptoms; (3) endorse cognitive reappraisal, anhedonia, brooding, and/or suicidal ideation rather than being someone who shows pronounced alpha asymmetry or engages in cognitive suppression.

Use of the putatively more specific PGS_MDD_ suggested four significant findings (i.e., for current MDD diagnosis, depressive symptoms, ERQ cognitive reappraisal, and brooding), but none survived correction for multiple testing. The results across both polygenic scores were largely consistent, apart from links with anhedonia and suicidal ideation, which were not significant in the PGS_MDD_ analyses, even prior to correcting for multiple testing. These findings are also interesting given that the PGS_MDD_ was defined in the UK Biobank using a much smaller sample of cases but a more specific sub-sample of those diagnosed with MDD as compared with the PGS_BD_.

However, the association between PGS_BD_ and suicidal ideation survived correction for multiple comparisons. The finding that the PGS_MDD_ was less sensitive for suicide-related phenotypes than the PGS_BD_ may underscore differences between the etiology of MDD and suicidal behavior. This might support the position that argues for suicidal behavior disorder to be considered a separate diagnostic entity in the DSM classification system^34^. The finding that the PGS_MDD_ was less sensitive for suicide-related phenotypes than the PGS_BD_ may underscore differences between the etiology of MDD and suicidal behavior. Anhedonia also differed between the broad and MDD PGSs. Recent evidence suggests that this core feature of depression may have distinct genetic and neuroimaging profiles compared with other features of depression^35^, so if this presumed dimension of depression was less prevalent in individuals identified with MDD in the UKB, the PGS_MDD_ might have less utility than the PGS_BD_.

Moreover, prior work has found an association between suicide attempts and a PRS for anhedonia—this association was observed even after controlling for an MDD PGS^36^. Together with the current study, this work suggests that anhedonia and suicidality may share a genetic etiology separate from MDD. Indeed, a recent meta-analysis finds that anhedonia and current suicidal ideation are robustly associated, even after controlling for concurrent depression^37^. This is consistent with the finding from the current study that the PGS_MDD_ was not strongly associated with either suicidal ideation nor anhedonia.

It was notable that the observed associations between the polygenic risk scores and the intermediate phenotypes for depression were quite small (the strongest β = 0.20 for suicidal ideation for the PGS_BD_). Our sample size of 210 had sufficient power (unadjusted) to detect an effect size that explained approximately 3.6% of the variance, which may have been overly optimistic. Indeed, after correcting for 8 statistical tests, we only had sufficient power to detect an effect size that explained approximately 5.8% of the variance. Others have pointed out that if PGSs account for 3% of the variance in a phenotype, sample sizes of approximately 300 are needed to achieve 80% power. Sample sizes of ≥ 800 would be needed for PGSs to account for 1% of the variance with even greater sample sizes needed to detect much smaller effects^38^. Given the small effects observed in the current study and previous work reviewed above, future work with depression-related PGSs would be well served to have 1000 participants or more. Nevertheless, this work provides useful guidance about the expected effect sizes for intermediate phenotypes for depression.

A somewhat atypical feature of this study is that we aimed to recruit a sample whose depression scores were normally distributed. This recruitment approach allowed us to examine the genetic contributions to depression-related phenotypes in a continuous manner, rather than comparing groups (e.g., high vs low in rumination). This approach should provide more statistical power than group-based analyses. Further, evidence suggests that many depression-related phenotypes differ in degree rather than kind^39^, another reason for recruiting participants in this manner.

Taken together the current study’s findings must be considered in light of several limitations. First, this study was conducted using summary statistics from GWAS of European Ancestry individuals, and the target sample was also comprised of European ancestry individuals. While this was done for both technical (e.g., using summary statistics from individuals with a similar genetic background to the target population produces more robust and accurate PGSs) and practical reasons (i.e., the clinical sample collected included EA individuals), it is still important to collect data and to examine these findings in non-European groups. Second, larger sample sizes assessed for depression-related measures might yield more significant findings in the future. That said, the patterns of results revealed in these analyses suggest that the amount of variance in depression-related phenotypes explained by UKB PGSs is relatively small. This is likely the result of several reasons that range from factors specific to the PGSs used, to an underwhelming transportability of PGSs that are seen across psychiatric and behavior genetics. Recent UKB analyses identified key differences in the genetic architecture between minimal phenotypes and more diagnostic phenotypes^18,33^. Specifically, by examining five depression phenotypes within UKB, they determined that SNP heritability of minimal phenotypes are lower than those for MDD and that use of a minimal phenotype identified genetic variation that was not specific to depression. Some possible explanations for this include power limitations even within consortia-based GWAS to date, a current reliance on linkage disequilibrium in GWA methods that does not identify functional variants, and the possibility that weights may be mostly sample-specific and the transportation of weights between samples impairs PGS performance. Efforts of large consortia, such as the Psychiatric Genetics Consortium, will provide key insights as to the origin of limited PGS transportability as they continue to aggregate ever larger samples for GWAS.

Future directions could include use of more diverse samples, larger samples to address power limitations, use of samples enriched for depression, and in other populations (e.g., developmental periods, sex-specific). As large-scale genomic consortia efforts (e.g., the Psychiatric Genetics Consortium, Million Veteran Program, All of Us) continue to increase in scale, additional polygenic scores will become available that may reflect a different depression phenotype than is currently available. Use of newly developed PGSs might yield different results than were seen here. Finally, examination of PGSs that index other forms of psychopathology, including personality disorders or neuroticism (explored in the supplemental materials section S1 of the present paper), may help highlight genomic variation that is depression-specific in contrast to genomic influences on psychopathology more generally.

In summary, broad and MDD-related PGSs derived from the UK Biobank accounted for small amounts of variance in eight depression-related phenotypes (i.e., MDD diagnosis, depression severity, alpha asymmetry, cognitive reappraisal, suppression, anhedonia, brooding, and suicidal ideation) characterized in an independent sample of adults. Only the association between PGS_BD_ and suicidal ideation survived correction for multiple comparisons in the current study. Nevertheless, these findings provide guidance about the expected effect sizes between current UKB PGSs for depression and depression-related neurocognitive phenotypes. These small effects suggest limited transportability of PGSs between large-scale efforts and smaller, intensively phenotyped samples. Future studies with improved power (both in the discovery and target datasets) may yield larger effects and increased utility.

## Methods

### Subjects

The protocol and procedures for the current study were ethically reviewed and approved by the Institutional Review Boards at the University of Texas and Emory University and all research was performed in accordance with the relevant guidelines and regulations. Phenotypic and genetic data were collected from 210 unrelated European ancestry adults recruited from the Austin, Texas community. As such, informed consent was obtained from all participants However, *N*s ranged from 206 to 210 depending on missing phenotypic data. Consistent with dimensional approaches to psychopathology^40^, participants were recruited along a continuum from no depressive symptoms to clinical levels to approximate a normally distributed sample of depression. Depression symptom severity was monitored during weekly project meetings via a Shiny app that extracted depression symptom severity data from the study database in real-time, plotted the distribution of the data, and then recruitment was adjusted as necessary. Most adjustments involved recruiting individuals at the higher end of the depression spectrum (i.e., screening out more participants with lower levels of depression as the study progressed). Recruitment was adjusted as needed to obtain a normal distribution of depression severity within the sample.

Participants were eligible if they met the following inclusion criteria: (1) 18–35 years of age; (2) European ancestry as accessed using principal component analysis and multi-dimensional scaling; (3) able to speak and read proficiently in English, and (4) either normal or corrected to normal vision. The exclusion criteria were: (1) current use of steroidal or psychotropic medications; (2) serious medical conditions; (3) heavy tobacco use defined as 20 cigarettes per day or greater than 20 pack years^41,42^; (4) a score of two or higher on the drug subscale of the Psychiatric Diagnostic Screening Questionnaire^43^; (5) a score of two or higher on the alcohol subscale of the Psychiatric Diagnostic Screening Questionnaire; (6) a score of one or higher on the psychosis subscale of Psychiatric Diagnostic Screening Questionnaire; or (7) being in imminent danger to others or self, or any recent suicidal behavior (suicidal ideation at level 4 on the Columbia-Suicide Severity Rating Scale in the past two months, or any suicidal behavior in the past two months).

Full participant demographics are reported in Table 1. Previous research with this sample examined associations between self-reported depression symptoms and negative cognitive biases^44^, identified predictors that reliably distinguish MDD, psychiatric controls, and healthy controls^45^, and used machine learning to identify neurocognitive predictors of reward responsivity^46^.

### Measures

The current study utilized a cross-sectional design with both genetic and phenotypic data collection. All phenotypes were residualized to adjust for age and sex and to transform variables to a more normal distribution.

Depression symptoms were measured with the following self-report questionnaires: Beck Depression Inventory-II (BDI-II)^47^ and the Snaith-Hamilton Pleasure Scale (SHAPS)^48^, a measure of anhedonia. Suicidal ideation question was taken from the BDI-II scale which had the following response options: (1) “I have thoughts of killing myself, but I would not carry them out”, (2) “I would like to kill myself”, or (3) “I would kill myself if I had the chance”.

Emotion regulation was measured with the brooding subscale of the Ruminative Response Scale (RRS)^49^, the Perseverative Thinking Questionnaire (PTQ)^24^, and the reappraisal and suppression subscales of the Emotion Regulation Questionnaire (ERQ)^50^.

Electroencephalography (EEG) was recorded during eight minutes of alternating eyes open and eyes closed at rest using a modified 64 channel montage BrainVision electrode cap and collected at a 500 Hz sampling frequency. Recording sites in the cap included standard and extended 10–20 system locations. Alpha power (8–13 Hz) was extracted and frontal alpha asymmetry was calculated by subtracting left from right log transformed EEG alpha power (ln_right_–ln_left_) at homologous frontal sites (i.e., F7/F8).

### Genotyping and quality control

Whole blood samples were stored in Dr. Beevers laboratory and transferred to Dr. McGeary’s laboratory for analysis. DNA was extracted from blood using QIAamp DNA Blood Maxi Kits (Qiagen, Valencia, CA) and DNA was extracted from saliva/buccal cells using manufacturers methods (Genotek, Ontario, Canada) and methods reported previously^51^. Extracted DNA was quantified and normalized per Illumina’s requirements for array genotyping (Picogreen and nanodrop). DNA was genotyped using the PsychArray BeadChip (Illumina).

Prior to imputing data, genetic variants with a genotyping rate less than 5%, rare variants (minor allele frequency < 1%), and individuals missing more than 10% of genetic data were removed. Datasets were then aligned to the Haplotype Reference Consortium (HRC) reference panel using a tool developed by the McCarthy Group^52^ that checked and updated marker information with respect to chromosome, base pair position, strand alignment, and reference alleles to match the HRC panel. Variants were removed if: (1) alleles were mismatched with the reference panel, (2) allele frequency differed by more than 0.20 from the reference panel, and (3) they were palindromic. Prior to imputation, a principal components analysis (PCA) was conducted using FlashPCA2 with the 1000Genomes reference panel, followed by multidimensional scaling, to identify individuals of European Ancestry. A second round of PCA was conducted within the European Ancestry subset to generate PCs to control for any residual stratification as done in prior work (Brick et al., 2019). Six PCs were conservatively selected based on visual inspection of scree plot (Supplemental Fig. S1) to be included as covariates in further analyses. Samples were then genetically imputed via Minimac4 genotype imputation software available on the Michigan Imputation Server, using the HRC r1.1 2016 admixed reference panel and Eagle v2.4 phasing^53^. Following imputation, variants were screened to only include biallelic variants located on autosomal chromosomes with an imputation quality score (*r*^2^) greater than 0.30. Additional post-imputation QC removed variants with a call rate < 95%, minor allele frequency < 1%, or failed the Hardy Weinberg Equilibrium test (p < 0.0001). After imputation and QC, the data contained 6,858,885 variants and up to 210 individuals with genetic and phenotypic data (*n* for each phenotype is presented in Table 1).

### Statistical analyses

In the interest of determining which facets of depression (broad vs. specific) are captured with our phenotypes, we employed summary statistics of two Depression GWAS in the UKB: Broad Depression and ICD-Coded MDD. Both Broad Depression and ICD-Coded MDD summary statistics originated from the same study^8^. Howard and colleagues defined broad depression as self-reported evidence of past help-seeking behavior for problems with “nerves, anxiety, tension or depression” meaning individuals who either have a primary/secondary diagnosis of a depressive mood disorder obtained from hospital records, or individuals who answered “yes” to the following questions at an assessment visit met criteria for broad depression: “Have you ever seen a general practitioner for nerves, anxiety, tension or depression?” or “Have you ever seen a psychiatrist for nerves, anxiety, tension or depression?”^8^. Summary statistics for Broad Depression included 7,641,986 variants from 113,769 cases that met criteria for Broad Depression and 208,811 controls in the UK Biobank (prevalence = 35.27%)^8^.

Cases for the GWAS of ICD-coded MDD were a subset of cases of Broad Depression, only including individuals that had either an ICD-9 or ICD-10 primary or secondary diagnosis for a depressive unipolar mood disorder (ICD codes: F32—Single Episode Depression, F33—Recurrent Depression, F34—Persistent mood disorders, F38—Other mood disorders and F39—Unspecified mood disorders)^8^. Summary statistics for ICD-coded MDD included 7,658,352 variants from 8276 cases that met criteria for ICD-coded MDD and 209,308 controls in the UK Biobank (prevalence = 3.80%)^8^.

Polygenic scores were calculated using the clumping and *p*-value thresholding method on variants that aligned across the Discovery GWAS^8^ and study datasets (number of *k* SNPs per trait were k_BD_ = 5,500,234; k_MDD_ = 5,510,487). We performed LD-clumping using PRSice^54^, to remove variants that were in Linkage Disequilibrium (i.e., using LD threshold (clump-*r*^2^) of 0.1, a physical distance threshold (clump-kb) of 250 kb and a p-value threshold (clump-*p*) of 0.05), which effectively removed redundant/correlated effects between variants^55–57^ (k_BD_ = 221,253; k_MDD_ = 222,509). Instead of testing multiple p-value thresholds, which would inflate the type I error rate, we used a *p*-value threshold of 0.05 to calculate the PGSs. A *p*-value of 0.05 was picked a priori for two reasons. First, multiple depression related phenotypes were tested, and it is likely that each phenotype would likely have a different *p*-value threshold that is the “best” predictor. Second, depression is known to be highly polygenic so a lower *p*-value may be too restrictive; however, including too many variants would likely introduce more noise than signal. This threshold reduced the number of SNPs contributing to each PGS (BD = 37,091; MDD = 31,376). PGSs were calculated using the effect size of each allele.

The effect of the Broad-Depression and MDD PGS (PGS_BD_ and PGS_MDD_, respectively) on the eight depression-related phenotypes was determined using multiple regression using maximum likelihood estimation in Mplus (version 8)^58^.Although not a primary focus of the study, post-hoc analyses also examined the effects of polygenic risk for neuroticism; details on PGS_Neuroticism_ are available in the supplementary text S1. All models included the first six genetic principal components (as determined appropriate by a scree plot) as covariates to account for SNP allele frequency differences across subpopulations within the data. We report standardized beta estimates as well as observed and corrected *p*-values adjusted for the eight correlated phenotypes within each sample using the P values adjusted for correlated tests (P_ACT_) method^59^. Phenotypic correlations among outcomes ranged from -0.46 for r_BDI-II-ERQ_reappraisal_ to 0.62 for r _BDI-II-Brooding_ (see Supplemental Fig. S2).

## Supplementary Information

Supplementary Information.

## Data Availability

The phenotypic and polygenic score data that support the findings will be available the Mood Disorders Laboratory Dataverse at [https://dataverse.tdl.org/dataverse/mdl?q=&types=dataverses;]. The data for the genotypes used to derive the polygenic scores will become available after an embargo period through dbGaP [https://www.ncbi.nlm.nih.gov/gap/], until that time, data may be made available upon request to the authors.
