# Supplementary material for: Associating broad and clinically defined polygenic scores for depression with depression-related phenotypes
Source: Sci Rep. 2023 Apr 21;13:6534. doi: 10.1038/s41598-023-33645-7 (PMC10121555; doi:10.1038/s41598-023-33645-7)
Supplement: Supplementary file 1 — Supplementary Information. [file 41598_2023_33645_MOESM1_ESM.docx]

**SUPPLEMENTAL ONLINE MATERIAL**

**Methods using PGS based on Neuroticism GWAS summary statistics**

Neuroticism is a personality trait often associated with risk for depression and other psychological disorders such as anxiety and schizophrenia ^1,2^. We use GWAS summary statistics from a study utilizing the UK Biobank sample ^3^. Their Neuroticism phenotype was created by summarizing a score of 12 neuroticism items (mood, mis, irr, hurt, fed-up, nerv-feel, worry, tense, worr-emb, suf-nerv, lone, guilt) ^3^.

*Calculation of Neuroticism PGS*

For Neuroticism, we employed the same methods used to create and test PGSs for broad depression and ICD-Coded MDD (see Methods section). First, duplicated, ambiguous, or mismatched variants were removed from the summary statistic data, as well as variants that were not present in the genetic data were removed from the summary statistic data, and only variants that matched between the summary statistic data and the genetic data were retained (k_Neuro_ = 5,568,91). Next, we performed clumping using PRSice, a wrapper of plink, to remove variants that were in Linkage Disequilibrium (LD) with each other, effectively pruning redundant correlated effects between variants ^4-6^. We then set the default values for clumping in PRSice: a LD threshold (clump-r2) of 0.1, a physical distance threshold (clump-kb) of 250 kb and a *p*-value threshold (clump-p) of 1 ^4^. Variants remaining after clumping were used in calculations of the PGS (k_Neuro_ = 212,900). To reduce the number of tests we performed, we used one p-value threshold of 0.05 to calculate the PGS (variants remaining after excluding *p*-value > 0.05: k_Neuro_ = 42,879). The PGS was calculated by taking the average effect size of each allele ^4^.

*Evaluating Performance of PGS*

In order to assess the ability of PGSs to explain genetic variance in the 8 depression-related phenotypes, we performed simple linear regressions via the software Mplus (version 8), using PGS_Neuroticism_ as the predictor, in conjunction with the six Principal Components as covariates (i.e., Y = β_0_ + β_1_ [PGS_Neuroticism_] + β_2_[PC1] + β_3_[PC2] + β_4_[PC3] + β_5_[PC4] + β_6_[PC5] + β_7_[PC6] + ε_i,_). Models were fitted in MPlus using maximum likelihood estimation ^7^. Variance explained in the model was determined using the R-squared test statistic and effect sizes of the PGS were determined using the standardized estimates.

*Summary of PGS_Neuroticism_ Effects*

Higher polygenic risk for neuroticism was associated with increased risk for suicidality (β=0.183 95% confidence interval [0.013, 0.353]; Supplementary Table 1) before correcting for multiple testing of correlated phenotypes. After correction effects were limited.

Supplementary Tables

| **Table S1**  ***Neuroticism Polygenic Score Effects on Depression and Related Phenotypes*** | | | | |
| --- | --- | --- | --- | --- |
| Phenotype | n | β [95% CI] | *p* (unadj) | *p* (adj) |
| MDD (current) | 208 | 0.096 [-0.077, 0.269] | 0.277 | 0.749 |
| Beck Depression Inventory - II | 210 | 0.078 [-0.095, 0.251] | 0.378 | 0.830 |
| Alpha Asymmetry (f7/f8) | 201 | -0.071 [-0.246, 0.104] | 0.426 | 0.670 |
| ERQ Cognitive Reappraisal | 210 | -0.064 [-0.237, 0.061] | 0.465 | 0.465 |
| ERQ Suppression | 210 | -0.106 [-0.273, 0.061 | 0.212 | 0.710 |
| SHAPS Anhedonia | 210 | 0.126 [-0.045, 0.297] | 0.149 | 0.610 |
| RRS Brooding | 210 | 0.095 [-0.078, 0.267] | 0.282 | 0.692 |
| BDI-II Suicide Item | 209 | 0.183 [0.013, 0.353] | 0.035 | 0.218 |
| *Note*. ERQ = Emotion Regulation Questionnaire; SHAPS = Snaith-Hamilton Pleasure Scale; RRS = Ruminative Response Scale; p (unadj) is the observed two-tailed p-value uncorrected for multiple testing with correlated outcomes. P (adj) refers to the false discovery rate adjusted p-value for correlated outcomes. | | | | |

Supplementary Figures


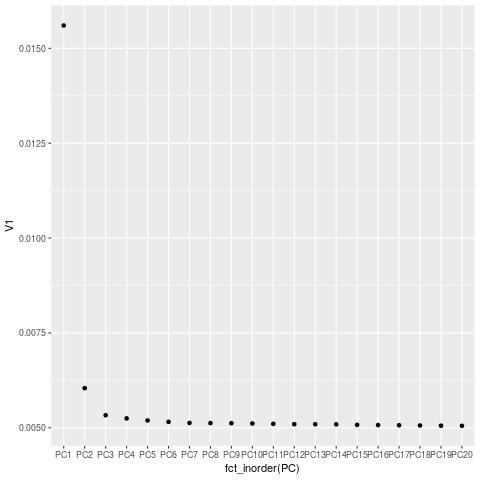


Supplementary Figure S1 Scree Plot of Principal Components

NOTE. The scree plot helped to determine the number of PCs to use as covariates.


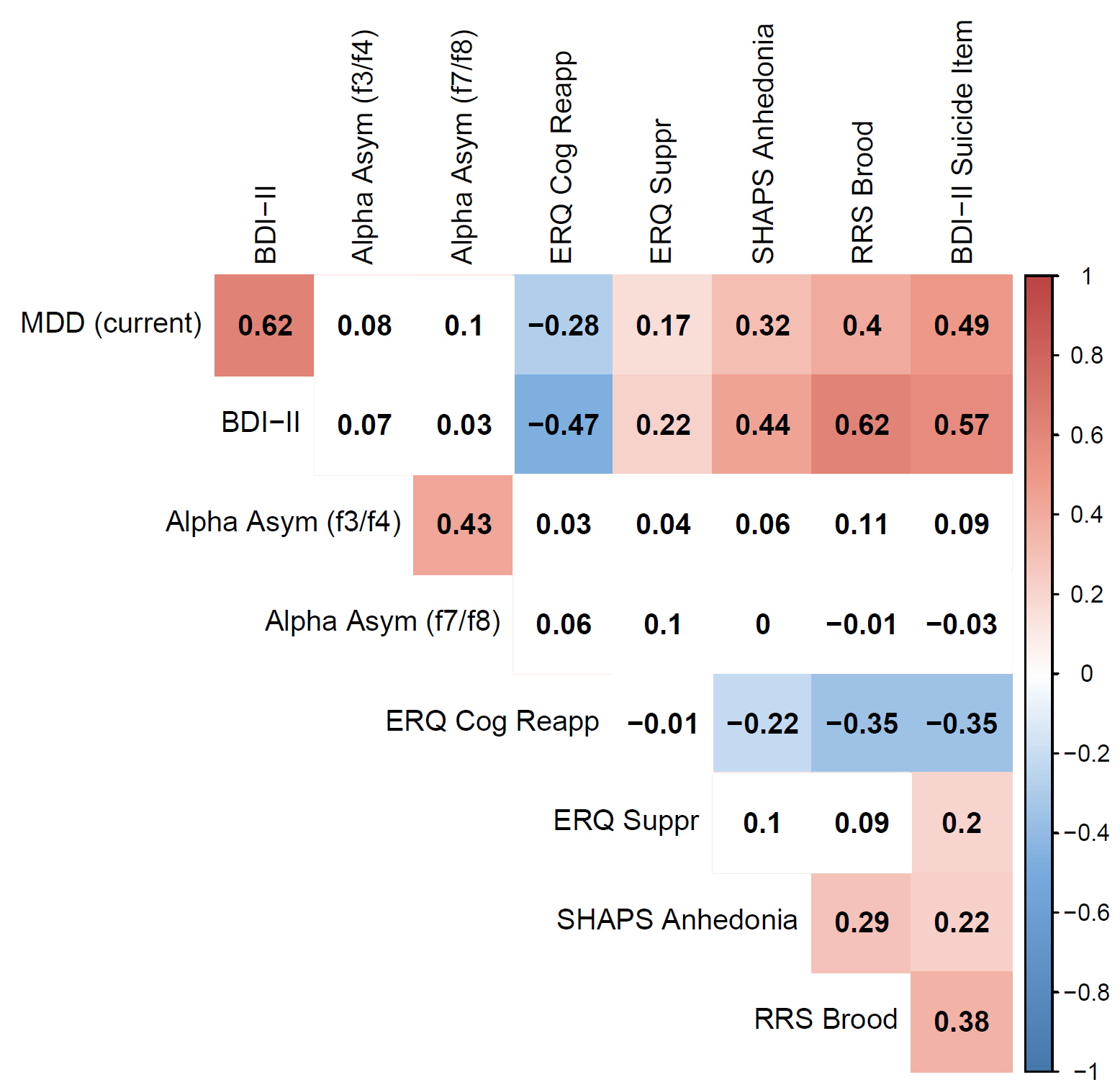


Supplementary Figure S2 Phenotypic Correlations

NOTE. non-white cells are significant p<0.05; Correlations were run on standardized residuals used in analyses, therefore Pearson’s r was used.

Supplementary References

1 Jardine, R., Martin, N. G. & Henderson, A. S. Genetic covariation between neuroticism and the symptoms of anxiety and depression. *Genet Epidemiol* **1**, 89-107, doi:10.1002/gepi.1370010202 (1984).

2 Adams, M. J. *et al.* Genetic stratification of depression by neuroticism: revisiting a diagnostic tradition. *Psychol Med*, 1-10, doi:10.1017/S0033291719002629 (2019).

3 Nagel, M., Watanabe, K., Stringer, S., Posthuma, D. & van der Sluis, S. Item-level analyses reveal genetic heterogeneity in neuroticism. *Nat Commun* **9**, 905, doi:10.1038/s41467-018-03242-8 (2018).

4 Choi, S. W. & O'Reilly, P. F. PRSice-2: Polygenic Risk Score software for biobank-scale data. *Gigascience* **8**, doi:10.1093/gigascience/giz082 (2019).

5 Chang, C. C. *et al.* Second-generation PLINK: rising to the challenge of larger and richer datasets. *Gigascience* **4**, 7, doi:10.1186/s13742-015-0047-8 (2015).

6 Prive, F., Vilhjalmsson, B. J., Aschard, H. & Blum, M. G. B. Making the Most of Clumping and Thresholding for Polygenic Scores. *Am J Hum Genet* **105**, 1213-1221, doi:10.1016/j.ajhg.2019.11.001 (2019).

7 Muthén, L. Mplus Users Guide. Los Angeles, CA: Muthén & Muthén; 2010. *Google Scholar*, 1-856 (2018).
